# Accurate detection for dental implant and peri-implant tissue by transfer learning of faster R-CNN: a diagnostic accuracy study

**DOI:** 10.1186/s12903-022-02539-x

**Published:** 2022-12-09

**Authors:** Woo Sung Jang, Sunjai Kim, Pill Sang Yun, Han Sol Jang, You Won Seong, Hee Soo Yang, Jae-Seung Chang

**Affiliations:** 1 Department of Artificial Intelligence, College of Engineering, Yonsei University, Seoul, Korea; 2 Department of Prosthodontics, Gangnam Severance Dental Hospital, College of Dentistry, Yonsei University, 211 Eonju-ro, Gangnam-gu, 06273 Seoul, Korea; 3 Graduate School of Public Policy, The University of Tokyo, Tokyo, Japan; 4 Department of Mechanical Engineering, College of Engineering, Yonsei University, Seoul, Korea

**Keywords:** Diagnostic imaging, Digital radiograph, Artificial intelligence, Deep learning, Peri-implantitis, Implant failure

## Abstract

**Background:**

The diagnosis of dental implants and the periapical tissues using periapical radiographs is crucial. Recently, artificial intelligence has shown a rapid advancement in the field of radiographic imaging.

**Purpose:**

This study attempted to detect dental implants and peri-implant tissues by using a deep learning method known as object detection on the implant image of periapical radiographs.

**Methods:**

After implant treatment, the periapical images were collected and data were processed by labeling the dental implant and peri-implant tissue together in the images. Next, 300 images of the periapical radiographs were split into 80:20 ratio (i.e. 80% of the data were used for training the model while 20% were used for testing the model). These were evaluated using an object detection model known as Faster R-CNN, which simultaneously performs classification and localization. This model was evaluated on the classification performance using metrics, including precision, recall, and F1 score. Additionally, in order to assess the localization performance, an evaluation through intersection over union (IoU) was utilized, and, Average Precision (AP) was used to assess both the classification and localization performance.

**Results:**

Considering the classification performance, precision = 0.977, recall = 0.992, and F1 score = 0.984 were derived. The indicator of localization was derived as mean IoU = 0.907. On the other hand, considering the indicators of both classification and localization performance, AP showed an object detection level of AP@0.5 = 0.996 and AP@0.75 = 0.967.

**Conclusion:**

Thus, the implementation of Faster R-CNN model for object detection on 300 periapical radiographic images including dental implants, resulted in high-quality object detection for dental implants and peri-implant tissues.

## Introduction

Panoramic and periapical radiography are necessary for long-term implant management while preventing and treating the corresponding complications [1]. While the panoramic radiograph confirms the overall oral condition, the periapical radiograph aids in the visualization of the gingiva, alveolar bone trabeculae, and the implant shape much more clearly owing to its high resolution [2]. Furthermore, the periapical radiograph is widely used as a method of examination for evaluating the biological and technical complications following implant treatment [3]. Using a periapical radiograph, the implant fixture shape and prosthesis can be confirmed, and appropriate treatment planning and prognosis can be derived by evaluating the level of resorption of the peri-implant marginal bone tissue [4–6].

However, there have been reports of varying results based on the resolution quality of periapical radiographs in measuring the resorption of the peri-implant marginal bone tissue [7]. Although the intra-and inter-examiner measurements were small, certain differences have been reported. Batenburg et al. [8] reported differences in the inter-observer error when measuring changes in the marginal bone surrounding several types of endosseous implants supporting mandibular overdentures following radiograph scanning. Moreover, Gröndahl et al. [9] reported 0.14 mm and 0.08 mm difference in inter-observer and intra-observer error, respectively, when measuring the radiographic bone level at Brånemark fixtures.

Thus, Artificial Intelligence (AI) could be efficient in handling measurements maintaining the same standards without such errors. For instance, based on the research on the implementation of deep learning in Computed Tomography (CT) images for pulmonary nodule detection in diagnosing lung cancer, the accuracy, sensitivity, and specificity of medical doctors diagnosing on 50 images of lung cancer were 79.6%, 81.3%, and 77.9%, respectively, whereas the deep learning model showed 92.0%, 96.0%, and 88.0%, respectively [10]. If AI could aid in the diagnosis of the level of changes in the peri-implant marginal bone tissue as well as gather information on the implant shape on periapical radiographs, AI would be able to aid in dental implants and peri-implant tissue detection in periapical radiographs.

Deep learning is being widely used in medical diagnoses [11–13]. In particular, various image data have been accumulated since digital imaging technology was adopted by the medical profession [14, 15]. Based on previous research reviews on deep-learning methods for medical image analysis, active collaboration has been ongoing between medical images/video data and vision-related deep learning, including classification, localization, detection, segmentation, registration, etc [16–19]. In particular, image classification, which is one of the vision-related fields in deep learning, is a project that classifies objects in an image based on various classes. For example, a classification of three classes of dental caries, periapical infection, and periodontitis was processed based on dental disease research [20].

A recent study, in which various classification models were applied to identify four types of different implants from the periapical radiographs, revealed findings similar to our study [21–24]. However, despite this achieved classification of various types of implants, there was a limitation wherein processing through deep learning for location detection was not possible which required cropping of the image for implant classification in the preprocessing stage. Therefore, by using the successful localization performance of dental implant and peri-implant tissue detection, efficiency of the classification model could be improved without the conventional pre-process of manually cropping the implant area. Moreover, the object detection field implemented in this study has been actively adopted by the medical profession. This is an automatic technique for detecting a target object from an image separated from the background [25]. For example, there is a study that detected cervical spinal cord injury and disc degeneration from 1500 cervical MRIs by using Faster R-CNN, which showed a mAP result of 0.886 [26].

Therefore, our study used periapical radiographs from periodic checkups following implant treatment to investigate the method of accurate detection of dental implants and peri-implant tissue together, using a deep learning model.

## Materials and methods

### Data collection

Intraoral X-rays (i.e. Planmeca intra, Planmeca, Helsinki, Finland) with a sensor (i.e. Fona CDRelite, Fona Dental, Assago, Italy) were used for the paralleling technique, and the exposure conditions were 63 kVp and 8 mA, with an exposure time of 0.125 s according to the manufacturer’s guidelines. Based on the dental hospital digital medical records, anonymous dental implant radiological imaging datasets were collected from January 2016 to June 2020.

In this study, a total dataset of 300 images of periapical radiographs of different patients was used, with 240 and 60 images as the training and test dataset, respectively; the total number of implants in the training and test dataset was 374 and 125, respectively.

This study complied with the EQUATOR guideline STARD 2015: An Updated List of Essential Items for Reporting Diagnostic Accuracy Studies and was approved by the Institutional Review Board (IRB No. 3-2020-0028). The study was designed to be non-invasive and retrospective, and all data were analyzed anonymously; hence, informed consent was not necessary. All data collection and experiments were performed according to approved ethical guidelines.

### Data preprocessing

Digital periapical radiographic images were obtained using PACS (Zetta PACS, TaeYoung Soft, Anyang-si, Korea) and then extracted to the PNG. The size was 640 × 900 or 900 × 640 pixels. Each radiographic image included various dental implants.

To proceed with the object detection task, the ground truth bonding box should be labelled, for which an opensource tool called ‘labelimg’ (github link: https://github.com/tzutalin/labelImg.git) was used. To reduce the manual noise during labeling, the crown, abutment, fixture, peri-implant soft tissue, and peri-implant marginal bone were all included in the bounding box, and the prosthodontist carefully processed the images by fitting the boundary line of the implant and peri-implant tissue as ground truth.

## Object detection model architecture

**Fig. 1:**
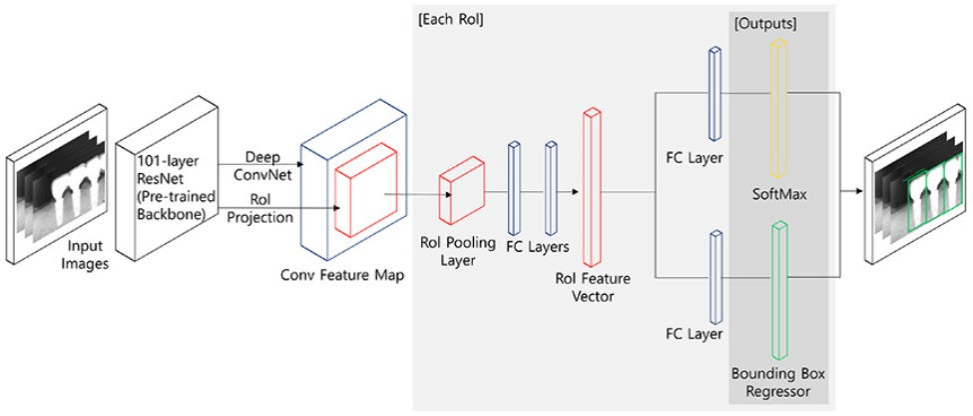
Faster R-CNN architecture applied to implant detection (with Resnet 101 backbone)

In this study, the Faster R-CNN model was applied, using Resnet 101 as the backbone [27]. Note that various classification models can be used for the pretrained CNN backbone of the object detection model. Faster R-CNN is an upgraded version of the previous fast R-CNN model [28]. In Fast R-CNN, selective search [29]. was used during region proposal, the process that detects locations where objects are likely to be found, and the RoI (Region of Interest) generation process acted as a bottleneck for this entire process and was calculated outside of the CNN. A region proposal network (RPN) was employed in the Faster R-CNN for integration of the RoI generation and CNN layer construction stages. Subsequently, sibling layers comprising a softmax output layer for classification and bounding box regressor layer resulted in a branching form after the following two processes: (1) the RoI pooling layer yielded a fixed length of feature vector from the region proposal, and (2) all network nodes were connected to form a Fully Connected (FC) layer. The softmax output layer predicts the object class, while the bounding box regressor layer predicts the location of an object that is responsible for classification and localization, respectively. The prediction outputs from these branches were compared with the class labels and bounding box coordinates of the ground truth labels, yielding the loss value. The model parameters were subsequently trained through back-propagation (Fig. 1).

### Model training

The Faster R-CNN used in this study was learned through back-propagation by calculating the loss functions composed against localization and classification, consistent with many deep learning models. A loss refers to the value calculated based on the difference between the prediction results of classification/localization and ground truth labels. The model used in this study also used back-propagation of the loss value within the neural network.

**Fig. 2:**
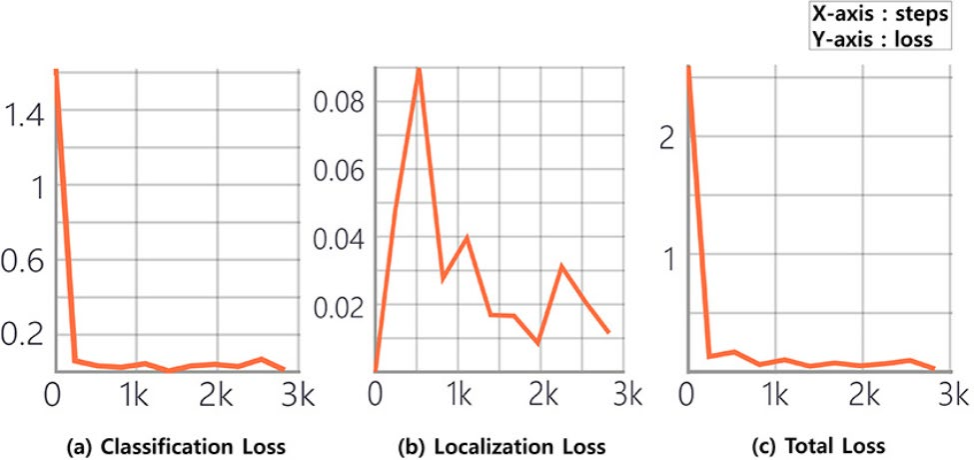
Training loss graph of the Faster R-CNN model for the 240 training datasets. (a) loss on object classification performance, (b) loss between the ground truth bounding box and the predicted bonding box, and (c) total loss of both classification and localization

In the learning process of Faster R-CNN, the term 1 epoch refers to one learning time on the whole train dataset, and this study tested the model weight that progressed 2826 times of steps in one batch unit. This is similar to approximately 12 epochs considering the trainset size of 240 images; since a convergence in the loss was demonstrated under such epoch, no further learning was processed. The loss in the object detection model presented a total loss, in which the classification and localization loss were combined demonstrating a decrease as the epochs progressed. The classification and total loss revealed an ideal graph with a minor fluctuation; however, it was relatively large in the localization loss (Fig. 2).

### Statistical analysis and evaluation metrics

Selecting evaluation indicators against the AI model is crucial; various metrics exist based on the type of task assigned by AI. Among these indicators, statistical indicators in the AI field, such as the comparison result for prediction (positive or negative) and ground truth, are widely used, consisting of four indicators: True Positive (TP), True Negative (TN), False Positive (FP), and False Negative (FN). Based on these four indicators, precision, recall, and F1 score, considering both, can be defined. These are widely used indicators in the performance evaluation of various AI models, including classification models (Eq. 1). In this study, precision, recall, and F1 score were used to evaluate the classification performance, excluding the localization of object detection.

1 $$\begin{array}{l} Precision = TP{\rm{ }}/{\rm{ }}\left( {TP{\rm{ }} + {\rm{ }}FP} \right),\\ Recall{\rm{ }} = {\rm{ }}TP{\rm{ }}/{\rm{ }}\left( {TP{\rm{ }} + {\rm{ }}FN} \right),\\ F1{\rm{ }} = {\rm{ }}2*\left( {Precision*Recall} \right){\rm{ }}/{\rm{ }}\left( {Precision{\rm{ }} + {\rm{ }}Recall} \right) \end{array}$$

**Fig. 3:**
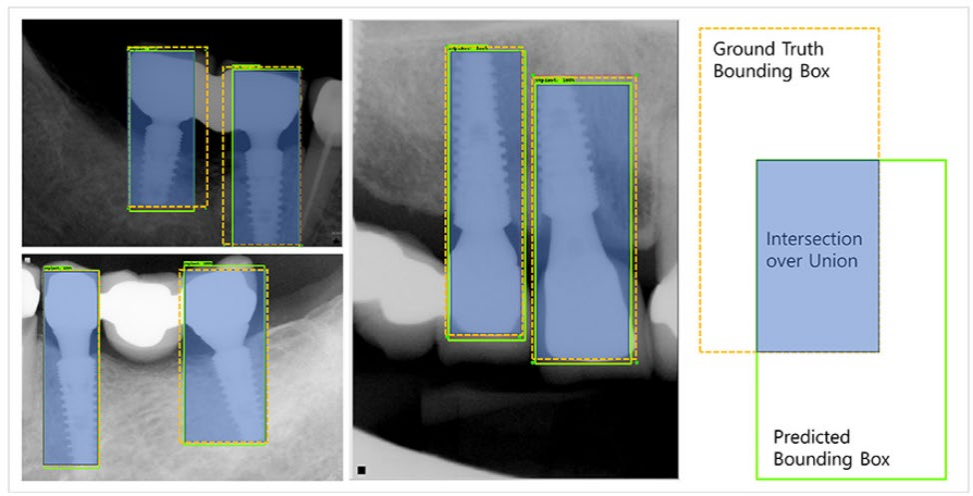
Schematic diagram showing the concept of IoU. The area where the ground truth bounding box and the predicted bounding box overlap indicates the IoU.

Meanwhile, as the object detection model carries out bounding box regression, Intersection over Union (IoU) is defined as an indicator of localization, which is the ratio of the overlapping area of the ground truth and the predicted area to the total area. Localization refers to predicting where the objects are located within an image (Eq. 2). For reference, the mean IoU shown in this study is defined as the calculated average of all IoUs from each test image used. Thus, this study examined the absolute minimum, absolute maximum, and average IoU (Fig. 3).

2 $$IoU{\rm{ }} = {\rm{ }}area{\rm{ }}of{\rm{ }}overlap{\rm{ }}/{\rm{ }}area{\rm{ }}of{\rm{ }}union$$

The Average Precision (AP), an integrated indicator comprising classification evaluation metrics and bounding box evaluation metrics shown above, is a widely used metric in the object detection field. The ‘$p$’ value in the equation refers to precision on the y-axis of PR (precision-recall) curve and the ‘$r$’ value refers to recall on the x-axis. (Eq. 3)

3 $$AP = \int\limits_0^1 {p(r)dr}$$

**Fig. 4:**
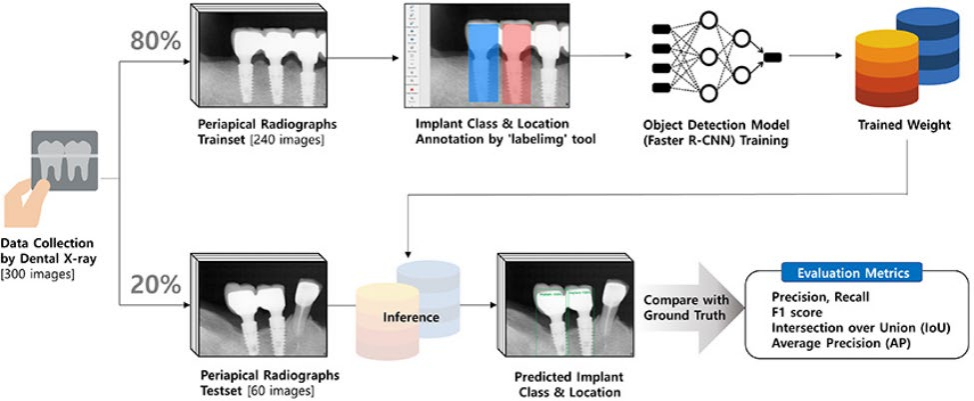
The strategy for data collection to data split, annotation and inference results, and evaluation metrics calculation process

Specifically, the model first collects prediction results of the detected objects and arranges them by rank based on the confidence level of the prediction. The results must simultaneously exceed the confidence score threshold. Next, IoU values are estimated to determine whether they exceed the IoU threshold (correct) or not (incorrect). These are accumulated in order of rank to calculate the precision and recall values, respectively, which are then used to construct a PR curve. Finally, the value obtained by integrating the area below this graph is the AP. AP@0.5 denotes the AP value when the IoU threshold is 0.5. Therefore, in our study, the result of AP@0.5, which is widely used, and the result of AP@0.75, measured using strict criteria, and the average value of AP@0.5 to AP@0.95 were compared. The results of deep learning object detection were analyzed using the PR curve (Fig. 4).

## Results

**Fig. 5:**
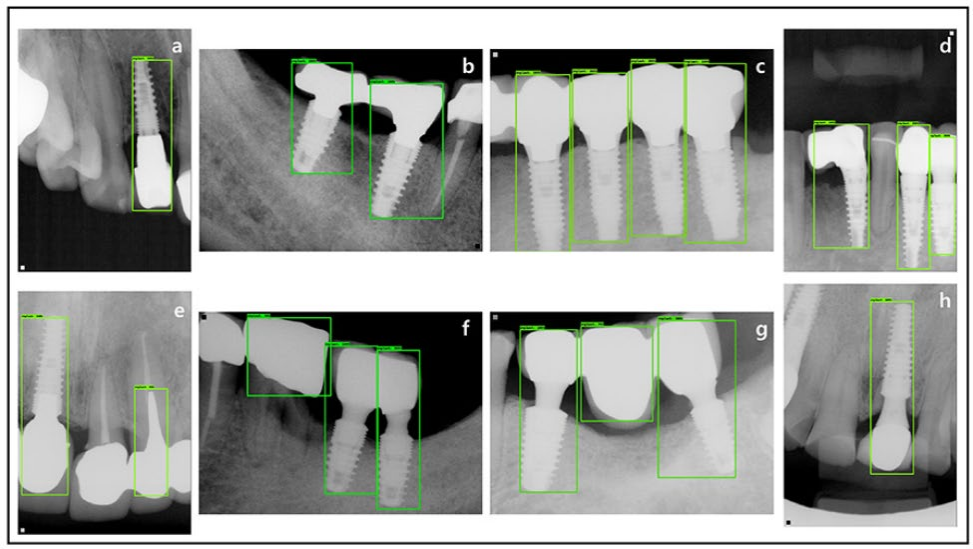
Example images of the result of the implant detection test. a–d: True positive (TP) cases that detect dental implants and peri-implant tissue well, e–g : False positive (FP) cases, h: false negative (FN) case

Various results of the running implant detection inference against the test dataset were obtained based on the trained detection model. The results showed TP = 124, FP = 3, and FN = 1 among all 125 ground truth labels, including all the dental implants and peri-implant tissue within the whole test dataset. Considering the FP and FN, the FN did not detect the implant profile that was truncated in the corner of the image. If all the profiles of the implant had been obtained clearly on the periapical radiograph, a higher accuracy would be anticipated. Meanwhile, FP occurred when brightness and saturation within the image were similar to those of implants such as crowns, pontics, and screw posts and crowns (Fig. 5).

First, considering the overall evaluation of the classification performance, based on the yielded TP, FP, and FN values, the precision, recall, and F1 score were calculated as 0.977, 0.992, and 0.984, respectively. These metrics evaluated the classification performance of dental implants and peri-implant tissue from the background. Furthermore, the localization performance could be considered precise, since the mean IoU value was 0.916 despite the various dental implant sizes and shapes and peri-implant tissue. Detailed statistics of IoU yielded a maximum IoU of 0.986, a minimum IoU of 0.640, and an IoU standard deviation of 0.050. In the metrics accounting for all classification and localization performances, AP@0.5 showed excellent performance of 0.996. Moreover, AP@0.75, which was evaluated on a stricter IoU threshold, also demonstrated a reasonable value of 0.967. On changing the IoU threshold by 0.05 from 0.5 to 0.95, average value AP@0.5:0.95 was derived as 0.849 (Table 1).

**Table 1:** Evaluated metrics of classification, localization, and object detection performance

Evaluation Target	Metrics	Results
Classification	Precision	0.977
	Recall	0.992
	F1 Score	0.984
Localization	Mean IoU	0.916
	Min / Max IoU	0.640 / 0.986
	IoU std	0.050
Object Detection	AP@0.5	0.996
	AP@0.75	0.967
	AP@0.5:0.95	0.849

**Fig. 6:**
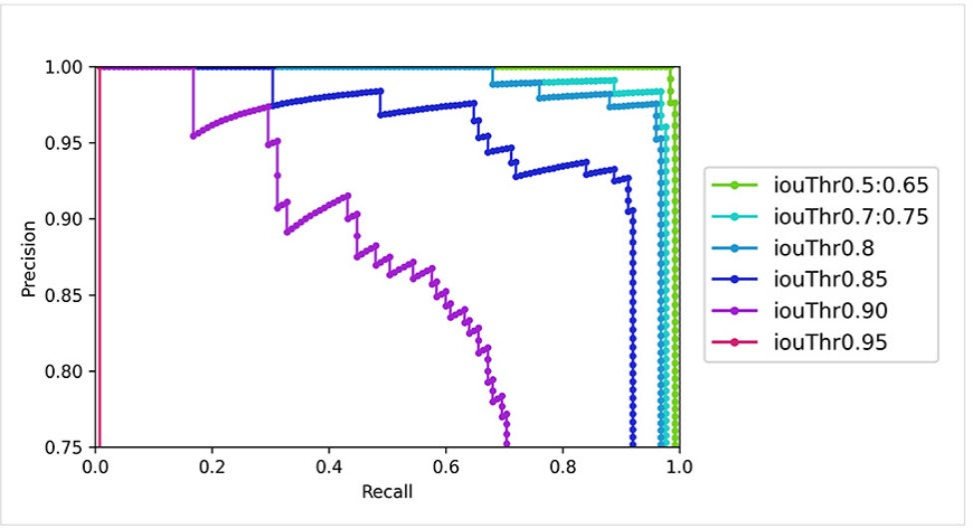
Changes in Precision-Recall curve according to intersection over union (IoU) thresholds variation

To obtain an AP, a PR curve must be derived, which is drawn differently based on the IoU threshold setting. For our test dataset, the same graph was drawn up to an IoU threshold of 0.5:0.65; the same graph is drawn up to 0.7:0.75, and different graphs are drawn every 0.05 IoU threshold increment thereafter. Following that, every time the IoU threshold is increased by 0.05, the trend of decreasing precision is more noticeable. In particular, when set to 0.95, the bounding box prediction for all the test sets does not exceed 0.95; hence, the graph is drawn to fall vertically (Fig. 6).

## Discussion

Many professionals believe that AI applications will gradually replace or substitute medical education for healthcare experts, especially in diagnosis [30]. For example, a study that used deep learning to predict exudative Age-related Macular Degeneration (AMD) demonstrated an average accuracy of 100% by DCNN in the diagnosis of Optos images (without a fundus examination), whereas the six ophthalmologists showed an average accuracy of 81.9% [31]. A similar result was obtained comparing the results of ameloblastomas and keratocystic odontogenic tumors made by CNN and oral maxillofacial specialists; however, CNN took 38 s and oral maxillofacial specialists took 23.1 min, demonstrating AI efficiency in diagnosis [32]. In our study, we applied theFaster R-CNN model and processed a comparative analysis to proceed with the implant detection task; consequently, it demonstrated accurate classification and localization performance for dental implants and peri-implant tissue, even with little data. It would be clinically beneficial to apply this model in evaluation of resorption level of the peri-implant marginal bone and identification of unknown implant in periapical radiograph.

Meanwhile, the mean IoU showed a fine result of 0.916 since Faster RCNN is a 2-stage model that has a separate RPN; hence, the location accuracy is relatively higher. This result was similar to the 0.91 of mean IoU, which was interpreted owing to the size and similarity of shape of the tooth and implant [33]. Specifically, the absolute minimum of IoU against 60 test images was 0.640, absolute maximum was 0.986, and standard deviation was 0.050. IoU values ​​are distributed around an average value, and the minimum IoU value is a result of the difference in localization of the cantilever part, as shown in (3) of Fig. 3. The slight difference in the IoU between the experts and the prediction was due to the slight difference of the area of the implant prosthesis and peri-implant tissue,reflecting an area of overlap; however, all parts of the fixture were included in the prediction area.

At the same time, during the process of AP calculation, based on the changes in the IoU threshold, we found that if the IoU threshold was set low, the classification performance improved while the localization accuracy decreased, whereas if set higher, the classification performance dropped while the localization accuracy improved [34]. In the results of AP, our Faster R-CNN showed high performance with AP@0.5: 0.996 and AP@0.75: 0.967. This was likely because the color distribution was monotonous and the implant thread had a clear geometrical feature, implying fine quality for practical use. In another study that applied an object detection model to dental implants, six implant systems were detected using Yolo v3 with a total of 1282 panoramic radiograph images [35, 36]. Although it is significant in that it detected a large number of implants, only the implant area containing no peri-implant tissue was detected, and the mAP of 0.71 was relatively insufficient in accuracy. This is mainly due to the fact that it used the panoramic radiograph, which has lower image quality compared to the periapical radiograph we used, and also due to the Yolo-type model that focuses on inference speed rather than accuracy. As such, before classifying various types of implants, we first conducted a study on how well a single implant and peri-implant tissue could be detected within the periapical radiograph and resulted remarkable performance for implant identification.

The faster R-CNN used in our study was developed quite a long time ago, and although it falls largely behind in terms of Frame Per Second (FPS) compared to recent object detection models; its accuracy remains competitive [37]. To apply Faster R-CNN to the implant detection task, we used TensorFlow object detection API (Application Programming Interface) [38]. Tensorflow provides detailed instructions, such that non-AI professionals, such as dentists, find it easily accessible and can customize the data. To improve the detection performance of the model in our study, we fine-tuned the implant dataset against the pretrained weight of the COCO (Common Objects in Context) image dataset [39]. This refers to a deep learning method called transfer learning, in which either the final model performance improves or the learning process becomes faster compared to learning from scratch [40]. Such a method is useful when there is a lack of data on new areas in medicine and dentistry. This is because neural networks have semantic information produced in the late CNN layers after patterns result from the early layers. Therefore, by changing the linear layers except for the early layers and performing fine-tuning, we could quickly and accurately perform the model study even with a small amount of data. We surmise that this is the reason for the good performance of our model despite a small amount of data.

Furthermore, although labeling has been processed based on the advice from professionals, the standard regarding the boundaries of the bounding box is still ambiguous and this limitation has been persistent in the field of object detection. Hence, applying image segmentation for categorizing geometrical features as the standard of various implants in future, we will enhance performance of multi-class implant detection.

## Conclusion

Object detection using the Faster R-CNN model against 300 periapical radiographic images, including implants, showed high performance in dental implant and peri-implant tissue detection.

## Data Availability

The datasets used and/or analyzed during the current study are available from the corresponding author on reasonable request.
